# Timing and efficacy of transjugular intrahepatic portosystemic shunt in patients with pyrrolizidine alkaloid-induced hepatic sinusoidal obstruction syndrome

**DOI:** 10.1038/s41598-021-01201-w

**Published:** 2021-11-05

**Authors:** Fan Wu, Jiao Yu, Hongying Gan, Heng Zhang, Deying Tian, Dan Zheng

**Affiliations:** 1 Department of Gastroenterology, The Central Hospital of Wuhan, Tongji Medical College, Huazhong University of Science and Technology, 26 Shengli Street, Jiang’an District, Wuhan, 430014 Hubei China; 2 Department of Gastroenterology, Tongji Hospital, Tongji Medical College, Huazhong University of Science and Technology, Wuhan, 430030 Hubei China

**Keywords:** Diseases, Gastroenterology

## Abstract

There is no specific treatment for pyrrolizidine alkaloid-induced hepatic sinusoidal obstruction syndrome (PA-HSOS). It is not clear when transjugular intrahepatic portosystemic shunt (TIPS) should be implemented in PA-HSOS patients. This study aimed to evaluate the timing of TIPS using total bilirubin (TBIL) as a measure, and to investigate efficacy of TIPS. We retrospectively analyzed the medical records of 10 PA-HSOS patients, among whom 4 patients had received TIPS (TIPS group), and the remaining patients were assigned to the internal medicine group. In the TIPS group, the TBIL level before TIPS was 84.4 ± 45.2 µmol/L (> 3 mg/dL), and TBIL levels were increased to different degrees after TIPS. With the extension of time, serum TBIL levels gradually decreased, and no liver failure occurred. With regards to the short-term outcomes, 3 patients recovered, 1 developed chronic illness and 0 died in the TIPS group. Moreover, 0 patients recovered, 5 developed chronic illness and 1 died in the internal medicine group. The rank sum test of group design revealed significant differences in clinical outcomes (P = 0.02). It was suggested that when the internal medicine effect of PA-HSOS patients is poor, TIPS should be considered, which is no trestricted to the limit of 3 mg/dL TBIL. It was also found TIPS effectively promote the recovery of liver function and reduce the occurrence of chronicity.

## Introduction

Hepatic sinusoidal obstruction syndrome (HSOS) is a clinical syndrome characterized by hepatomegaly, ascites and hyperbilirubinemia^1^, and is characterized by the pathological features of sinusoidal endothelial damage^2^. In developed countries, HSOS mostly occurs after bone marrow hematopoietic stem cell transplantation (HSCT) pretreatment^3^. In China, the main cause of HSOS is the intake of herbs or dietary supplements containing pyrrole alkaloid (PA)^4,5^. The incidence of PA-associated HSOS (PA-HSOS) caused by herbs containing PA, such as Tusanqi, is increasing^6^. PA-HSOS typically manifests as liver function damage and portal hypertension, and its diagnosis is mainly based on non-specific clinical features and invasive liver biopsy^7^. Moreover, the exposure to PA is highly important for the diagnosis of PA-HSOS. In terms of treatment, most studies focus on HSCT-induced HSOS (HSCT-HSOS) rather than PA-HSOS^8^. The standard therapy includes symptomatic supportive treatment of PA-HSOS, involving hepatoprotection, diuretic therapy and improvement of the microcirculation, and anticoagulant therapy is recommended unless contraindicated. If the afore mentioned treatment is ineffective, transjugular intrahepatic portosystemic shunt (TIPS) and liver transplantation should be considered. However, current evidence is limited and there is a lack of verification. Moreover, there is no specific treatment for PA-HSOS that has been globally recommended^4^. These afore mentioned issues may compromise the diagnosis and treatment of PA-HSOS, thus, PA-HSOS has a high mortality rate^9^.

TIPS is an effective method for reducing portal pressure^10^, but TIPS can cause liver ischemia, which can lead to liver failure. Current guidelines do not recommend TIPS for patients with Child–Pugh scores ≥ 14 and end-stage liver disease (MELD) scores ≥ 19^11,12^. In 2018, the European Association for the Study of the Liver (EASL) published clinical practice guidelines for the management of patients with decompensated cirrhosis, and these guidelines clearly state that TIPS is not recommended in patients with serum total bilirubin (TBIL) levels of > 3 mg/dL^13^. The pathology of PA-HSOS includes swelling, damage, and shedding of hepatic sinusoid endothelial cells in hepatic acinus zone III and significant dilation and congestion of hepatic sinusoids^4^. These pathological changes can cause liver damage and portal hypertension, patients often present with jaundice, ascites and so on.

For patients with poor symptomatic supportive treatment effect, it remains unclear when TIPS should be performed in patients with PA-HSOS. Due to the early administration of TIPS, patients may spend lots of money, and there may be some adverse events may occur during and after TIPS. If TIPS intervention is delayed and liver function deteriorates, there is no opportunity for TIPS. Therefore, the present study enrolled PA-HSOS patients as the research subjects, and investigated the timing of TIPS in PA-HSOS patients, using serum TBIL as a measure. We also observed the clinical outcome of the patients with PA-HSOS. It was hoped to provide a treatment reference for patients with PA-HSOS.

## Results

### Patient characteristics

**Figure 1:**
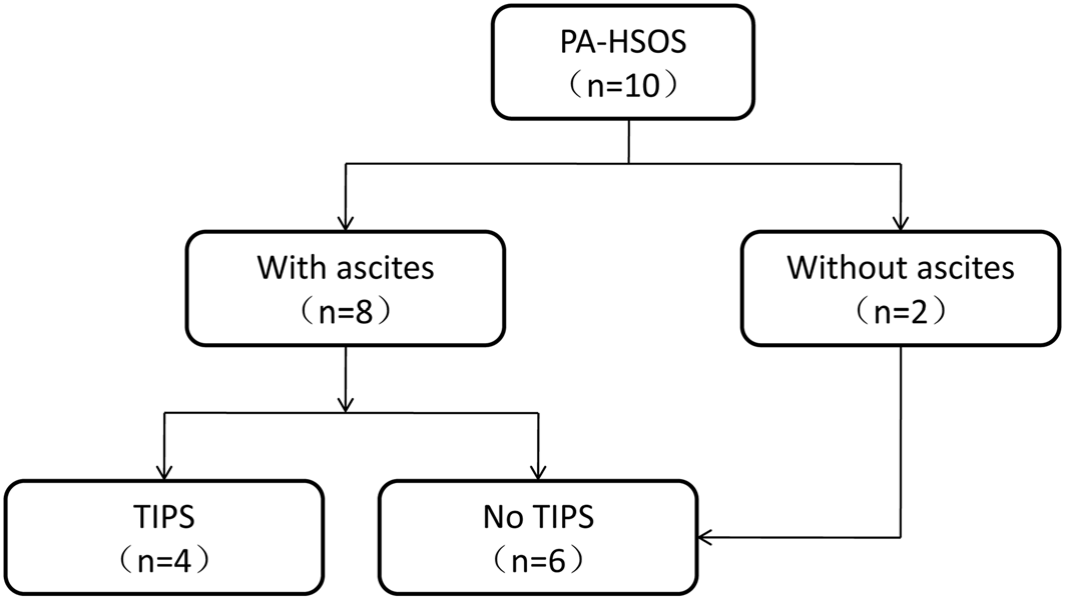
Flow chart of the 10 patients.

In total, 10 patients were included in the study, of whom 4 received TIPS treatment (Fig. 1). There was no significant difference in age, gender, red blood cell (RBC), hemoglobin (HB), platelet (PLT) count, alanine aminotransferase (ALT), aspartate aminotransferase (AST), TBIL, gamma-glutamyl transpeptidase (GGT), ALB, prothrombin time (PT), international normalized ratio (INR), ascites, hepatomegaly, splenomegaly, Child–Pugh scores and end-stage liver disease (MELD) scores between the TIPS group and the internal medicine group (Table 1). The clinical characteristics of patients were assessed. First, the results of blood routine examination showed that the PLT value was in the normal range in most of the PA-HSOS patients. Second, the results of liver function tests showed that biomarkers of liver damage (ALT, AST) and cholestasis (TBIL, GGT) exceeded the upper limit of normal range in most of the HSOS patients, while ALB exhibited different degrees of reduction. Third, INR was normal in most PA-HSOS patients. Moreover, based on the CT examination, the incidences of ascites, hepatomegaly and splenomegaly were 80%, 100% and 0%, respectively.

**Table 1:** Baseline characteristics of patients with PA-HSOS.

Variable	Case1	Case2	Case3	Case4	Case5	Case6	Case7	Case8	Case9	Case10
Age, years	48	49	71	59	25	73	58	56	48	61
Gender (M/F)	F	M	M	M	M	F	F	F	F	F
RBC, 10^12^/L	4.8	4.7	5.3	3.8	2.9	3.6	4.3	5.3	2.8	5.0
HB, g/L	128	141	164	125	102	110	139	165	89	135
PLT, 10^9^/L	204	142	86	229	49	84	124	103	249	136
ALT, U/L	21.3	12.8	76	164.8	169.1	40.4	50.5	44.7	21.5	29.8
AST, U/L	34.6	48	88	197	335	68.4	55.3	63.5	31.5	52.5
TBIL, μmol/L	16.9	90.7	88.3	463.2	346.5	22.4	43.7	71.3	47.9	101.5
GGT, U/L	135.6	214.2	77.7	284.2	1043.6	52.0	78.1	78.2	732.7	85.0
ALB, g/L	29.3	30.4	34.2	37	20.6	29.5	39.8	33.2	31.4	3.1
PT, S	11.1	13.6	18.9	11	14.2	15	17	15.5	12.6	15.1
INR	0.94	1.18	1.67	0.93	1.21	1.25	1.46	1.31	1.1	1.3
BUN, mmol/L	5.2	4.8	7	5.2	8.9	5.8	7.3	4.6	7.3	11.3
Cr, μmol/L	57.5	65	76.2	80	143.1	63.8	101.9	56.2	84	106.6
Ascites	+	−	+	−	+	+	+	+	+	+
Hepatomegaly	+	+	+	+	+	+	+	+	+	+
Splenomegaly	−	−	−	−	−	−	−	−	−	−
Child–Pugh scores	6	8	11	7	10	8	7	10	9	10
MELD score	6	15	18	19	25	10	16	15	11	18

*PA-HSOS* pyrrolizidine alkaloid-induced hepatic sinusoidal obstruction syndrome, *F* female, *M* male, *RBC* red blood cell, *HB* hemoglobin, *PLT* platelet, *ALT* alanine aminotransferase, *AST* aspartate aminotransferase, *TBIL* total bilirubin, *GGT* gamma-glutamyl transpeptidase, *ALB* albumin, *PT* prothrombin time, *INR* international normalized ratio, *BUN* blood urea nitrogen, *Cr* creatinine, *MELD* end-stage liver disease.

### Radiological findings of CT in PA-HSOS patients

**Figure 2:**
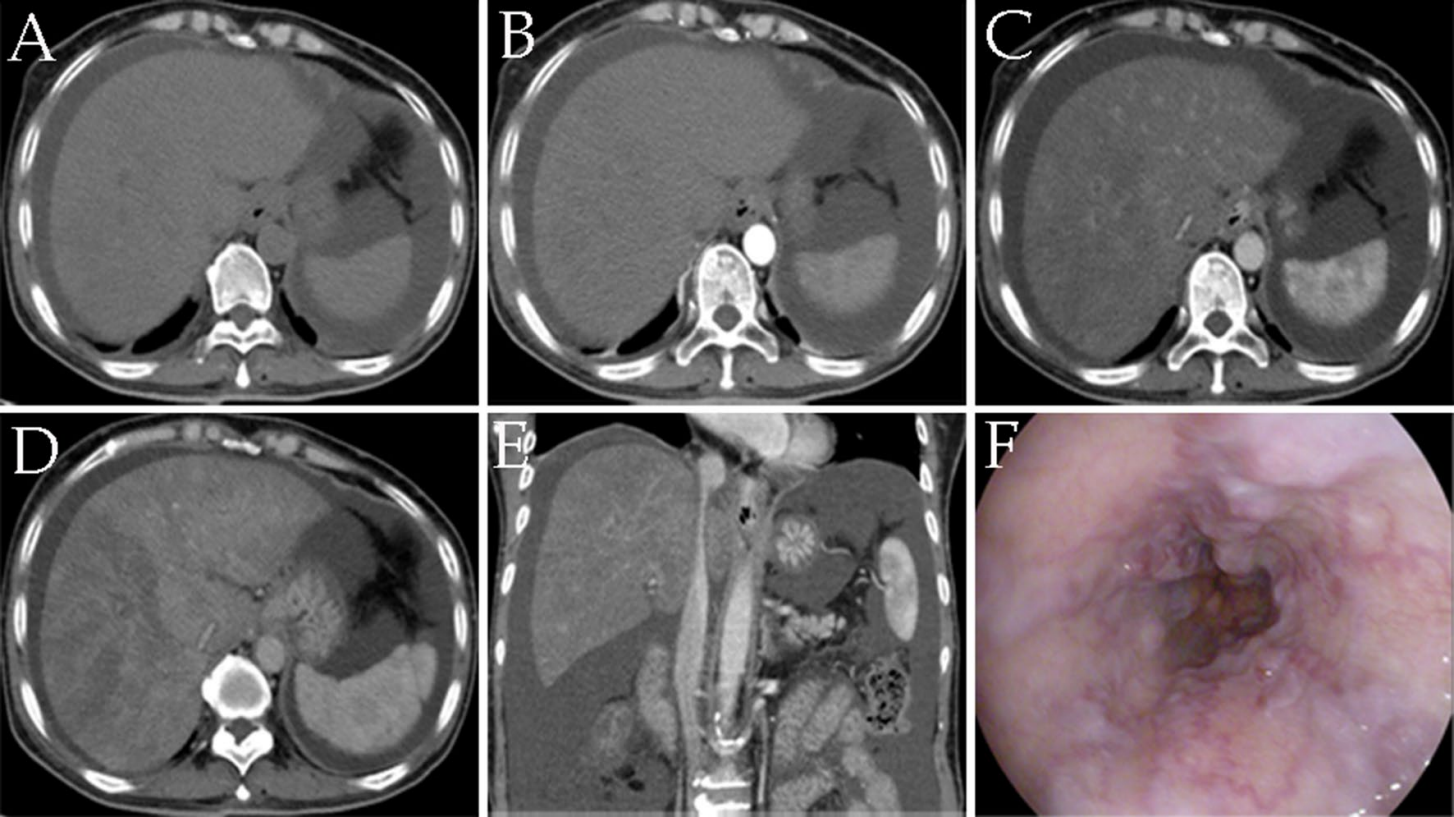
A 61-year-old woman diagnosed with pyrrolizidine alkaloid-induced hepatic sinusoidal obstruction received contrast-enhanced CT scan and gastroscopy. (**A–D**) Images from plain and contrast-enhanced CT scan. (**A**) Plain CT scan; (**B**) arterial phase; (**C**) portal phase; (**D**) equilibrium phases. (**E**) Multiple planar reconstruction CT showed that the hepatic segment of the inferior vena cava was compressed and thinner. (**F**) Gastroscopy revealed esophageal varices. *CT* computed tomography.

We analyzed the findings on plain CT and contrast-enhanced CT in the patients with PA-HSOS. Plain CT confirmed the presence or absence of ascites, hepatomegaly and splenomegaly, but these were not specific. Contrast-enhanced CT examination revealedan uneven enhancement of the entire liver and heterogeneous hypoattenuation. Typical CT images are shown in Fig. 2. In addition to CT images, in patients receiving TIPS, gastroscopy identified mild esophageal varices, which indirectly indicated the presence of portal hypertension.

### Changes of the portal vein blood flow in patients receiving TIPS treatment

**Figure 3:**
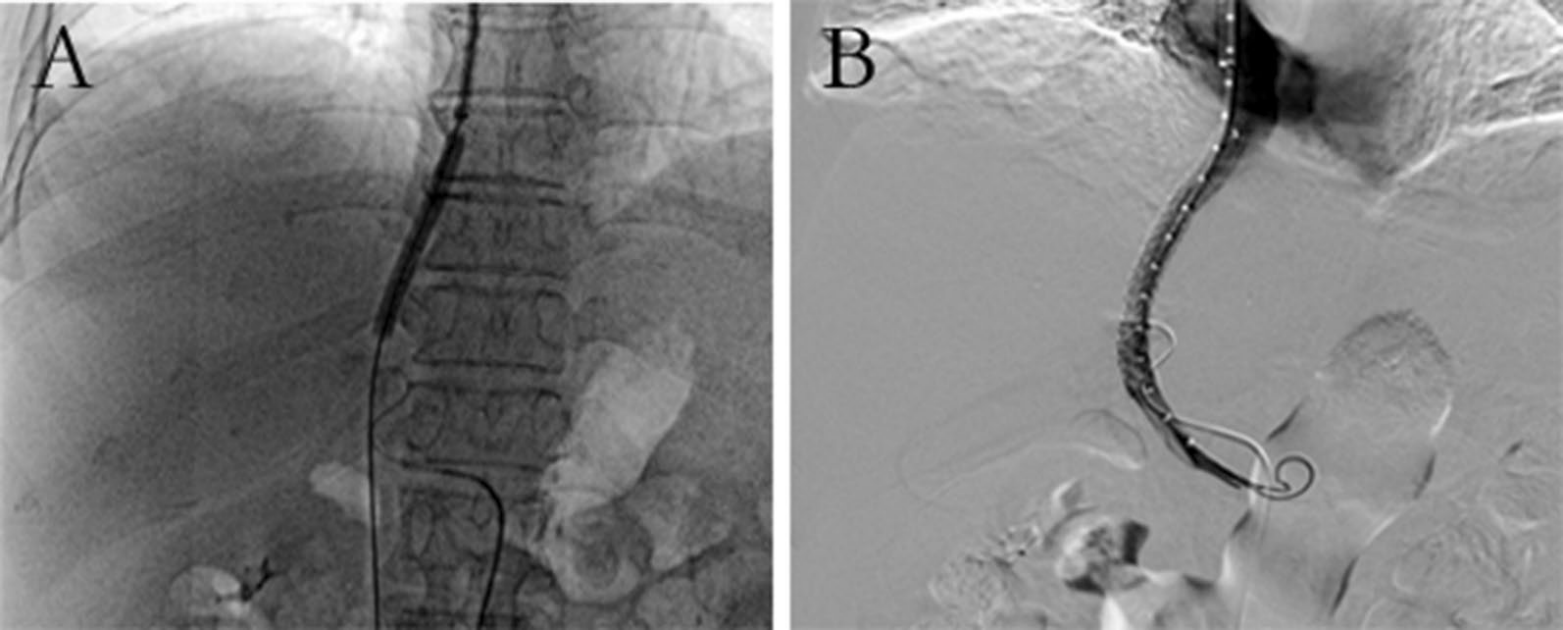
Digital subtraction angiography images from a 61-year-old female patient receiving transjugular intrahepatic portosystemic shunt treatment. (**A**) After successful puncture of the portal vein, the puncture tract was expanded with a balloon. (**B**) Portal vein angiography image after portal vein stent implantation.

TIPS surgery was successfully performed in 4 patients. After successfully puncturing the portal vein through the jugular vein, balloon catheter was used to dilate the hepatic parenchymal tract (Fig. 3A). After the stent was released, portal vein angiography was performed. The following observations were made: part of the portal vein blood directly returned to the heart through the stent (Fig. 3B).

### Serum TBIL change before and after TIPS

**Figure 4:**
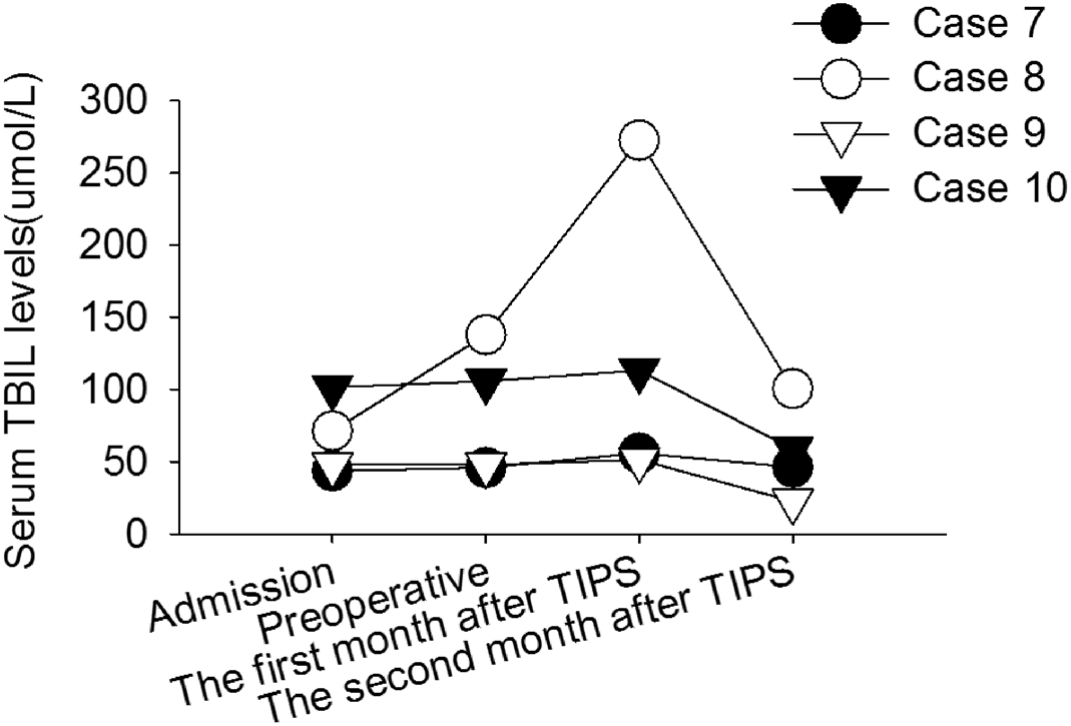
Changes of TBIL levels in patients receiving TIPS. Changes of serum TBIL levels in patients with PA-HSOS receiving TIPS. The patients exhibited varying degrees of elevation in TBIL levels after TIPS, indicating there was no liver failure. *TIPS* transjugular intrahepatic portosystemic shunt, *TBIL* total bilirubin, *PA-HSOS* pyrrolizidine alkaloid-induced hepatic sinusoidal obstruction syndrome.

Serum TBIL levels at admission and before TIPS intervention (+ 0 to 2 days) were recorded in 4 patients. The maximum concentration of serum TBIL was also reportedin the first month and the second month after TIPS (Fig. 4). The TBIL level of 4 patients before TIPS intervention was 84. 4 ± 45. 2 µmol/L, and the TBIL levels of 2 of these patients were > 51. 3 µmol/L (3 mg/dL). The serum TBIL levels of all 4 patients increased to varying degrees after TIPS. It was found that, with the extension of time, the serum TBIL levels gradually decreased.

### Clinical outcome of patients with PA-HSOS

In total, 10 patients were followed up until December 31, 2019. In the TIPS group, the ascites disappeared in 3 patients within 2 months, while the specific time of the ascites disappearance of 1 case was unknown. With regards to the short-term clinical outcomes, 3 patients recovered, 1 developed chronic illness and 0 died in the TIPS group. Moreover, no patients recovered, 5 developed chronic illness and 1 died in the internal medicine group (Table 2). The rank sum test of group design showed significant differences in the short-term outcomes between the TIPS group and the internal medicine group (P = 0.02).

**Table 2:** Outcome of patients with PA-HSOS.

	n	Recovery	Chronicity	Death
TIPS group	4	3	1	0
Internal medicine group	6	0	5	1

*PA-HSOS*, pyrrolizidine alkaloid-induced hepatic sinusoidal obstruction syndrome, *TIPS*, transjugular intrahepatic portosystemic shunt.

## Discussion

For PA-HSOS patients with poor response to internal medicine treatment, knowing when to select TIPS has been challenging for clinicians. The present study found that, when the efficacy of internal medicine treatment in patients with PA-HSOS was poor, i.e., when patients with ascites were difficult to treat with conventional treatment, with or without significant liver damage, TIPS should be considered, which is not restricted to the limit of 3 mg/dL serum TBIL. TIPS could effectively reduce the pressure of the portal vein, which was conducive to the resolution of ascites and the reduction of the occurrence of chronic diseases. The current study provided a reference for the clinical treatment of PA-HSOS patients. However, a limitation of this study was that the number of PA-HOS cases was small and the follow-up observation time was short.

The first step of treatment for PA-HSOS patients is to discontinue the intake of PA-containing plants, and then the patient is given medical treatment. If medical treatment is ineffective, the patient may progress to liver failure, death or chronicity. Furthermore, chronic patients may have long-term liver dysfunction. When the patient’s ascites subsides unsatisfactorily and liver damage continues to aggravate, TIPS should be used to significantly improve the patient’s portal hypertension and refractory ascites^4^. TIPS can effectively reduce portal pressure, and to a certain extent, it can improve the liver congestion state of PA-HSOS patients, which is beneficial to the recovery of liver function and the regression of ascites. However, whether TIPS can improve long-term prognosis requires longer follow-up observation^14^. Regarding the timing of TIPS intervention, in addition to refractory ascites which can be used as an indicator of intervention time, the liver function status of the patient should be referred to. To a certain extent, the concentration of TBIL represents the degree of liver function. For example, the higher level of the TBIL, the more severe the liver damage, and the risk of liver failure is greater in these patients after TIPS. Abnormal liver function was observed after examination in most patients with PA-HSOS^6^. The guideline published by EASL clearly stated that TIPS was not recommended in patients with decompensated cirrhosis with serum TBIL levels of > 3 mg/dL^13^. The guidelines for PA-HSOS published by China recommend that, in patients who do not respond adequately to medical treatment, TIPS can be used to control refractory ascites and portal hypertension, following careful weighing of the benefits against therisks^4^. However, this guideline has no clear recommendation on the TBIL level for TIPS. Peng et al.^15^ observed the efficacy of TIPS in the treatment of PA-HSOS patients, but did not study the timing of TIPS intervention, and did not show the serum TBIL level of patients before TIPS. In our study, 4 patients received TIPS for refractory ascites associated with portal hypertension. The TBIL levels of the 4 patients all increased to varying degrees after TIPS. With the extension of time, the TBIL levels gradually decreased, and no liver failure occurred after TIPS. For PA-HSOS patients with intractable ascites, with or without continued elevation of TBIL (TBIL > 3 mg/dL), TIPS should be considered to reduce portal pressure, which can improve the patient’s liver congestion and help restore liver function, and these factors may be more important than the potential occurrence of liver failure. Based on the findings of the present study, when internal medicine effect of PA-HSOS patients is poor, TIPS should be considered, which cannot be restricted to the limit of 3 mg/dL serum TBIL.

With regards to the efficacy of TIPS in the treatment of HSOS, Senzolo et al.^16^ revealed that the survival rate of HSCT-HSOS patients undergoing TIPS treatment was 20%, and if the patient had combined multiple organ failure, TIPS was performed too late. In a retrospective study, it was found that TIPS improved the prognosis of 29 PA-HSOS patients who did not respond to internal medicine treatment^17^. However, in another study, TIPS did not change the prognosis of patients with PA-HSOS^18^. In the current study, 4 patients received TIPS treatment, and the patients had a favorable outcome. Medical treatment, TIPS and liver transplantation are the three main aspects of treatment for PA-HSOS patients. Based on the present findings, TIPS should be considered when the effect of medical treatment is poor, and TIPS should not be restricted to the limit of 3 mg/dL TBIL. Unfortunately, the number of cases in this study was small, and the findings should be further verified in a study using a larger sample. Nevertheless, the present study can provide a reference for the treatment of patients with PA-HSOS.

## Methods

### Patients selection

This retrospective study was approved by the Ethics Committee of The Central Hospital of Wuhan [approval no. (2021)002], and the requirement for informed consent was waived. All methods were carried out in accordance with relevant guidelines and regulations. In total, 10 PA-HSOS inpatients from our hospital between March 2017 and July 2019, with a clear history of exposure to Tusanqi, were observed. The diagnosis of PA-HSOS was made according to the ‘Nanjing criteria’^4^, i.e., a history of PA exposure and three of the following: Abdominal distention, painful hepatomegaly or ascites; hyperbilirubinemia or abnormal liver function indicators; and typical features on plain computed tomography (CT) and contrast-enhanced CT. The following diseases were excluded: Viral hepatitis, autoimmune liver disease, alcoholic liver disease, Budd-Chiari syndrome, liver tumor and heart failure.

### Procedures

In total, 10 patients underwent the following examinations: Routine blood evaluation, liver function assessment, detection of viral hepatitis markers, plain CT and contrast-enhanced CT, ascites ultrasound, amongst others. The internal medicine treatment methods included hepatoprotection (reduced glutathione, ursodeoxycholic acid), anticoagulant therapy (low-molecular-weight heparin sodium or warfarin), correction of hypoproteinemia [human serum albumin (ALB) injection] and diuretic therapy (spironolactone and furosemide). However, the treatment efficacy was not obvious, meaning that serum TBIL levels were not effectively reduced, with or without refractory ascites. In total, 4 patients received TIPS treatment according to the wishes of the patients and their families (the TIPS group), while 6 patients continued to be treated with the internal medicine treatment methods (the internal medicine group). The general steps of TIPS were as follows: The portal vein was punctured through the jugular vein. After successful puncture of the portal vein, the puncture tract was expanded with a balloon. An 8-mm diameter VIATORR stent graft (W. L. Gore & Associates, Inc., Flagstaff, AR, USA) was implanted between the portal vein and hepatic vein. The specific length of the stent varied from patient to patient. Specific surgical procedures are detailed in the previous literature^9^.

After discharge from the hospital, the patients received a clinical evaluation every 1–2 months for the first 6 months, and then every 6 months thereafter. The clinical end points included death and liver transplant. The observation time was up to December 31, 2019.

### Data collection

Patient data during hospitalization were collected, including sex, age, routine blood tests, biochemistry, gastroscopy and imaging findings, amongst others. After discharge from the hospital, the patient’s liver function, color Doppler ultrasound and other test data were also recorded.

### Statistical analyses

The measurement data were expressed as the mean ± standard deviation. When comparing the baseline characteristics, a two-sample t-test was used to test whether the means of two groups were equal. A Chi-squared test and two-tailed Fisher’s exact test were used to compare associations. A rank sum test was used to test for disease outcomes. All tests were performed using SPSS 17.0 software (SPSS, Inc., Chicago, IL, USA). P < 0.05 was considered to indicate a statistically significant difference.

## Supplementary Information

Supplementary Information.

## Data Availability

All data generated or analysed during this study are included in this published article (and its Supplementary Information files).
