# Supplementary material for: Timing and efficacy of transjugular intrahepatic portosystemic shunt in patients with pyrrolizidine alkaloid-induced hepatic sinusoidal obstruction syndrome
Source: Sci Rep. 2021 Nov 5;11:21743. doi: 10.1038/s41598-021-01201-w (PMC8571302; doi:10.1038/s41598-021-01201-w)
Supplement: Supplementary file 1 — Supplementary Information. [file 41598_2021_1201_MOESM1_ESM.pdf]

Supplementary Materials Catalogue

1. Baseline characteristics and comparison------------------------------- page2

2. Raw data for Figure 4-- page2

3.Interventional data of 4 patients receiving TIPS------------------- --- page4

Case7---page4

Case8---page6

Case9---page8

Case10---page10

Baseline characteristics and comparison

	Ages	gender	RBC	HB	PLT	ALT	AST	TBIL	GGT	ALB	PT	INR
case1	48	female	4.82	128	204	21.3	34.6	16.9	135.6	29.3	11.1	0.94
case2	49	male	4.71	141	142	12.8	48	90.7	214.2	30.4	13.6	1.18
case3	71	male	5.26	164	86	76	88	88.3	77.7	34.2	18.9	1.67
case4	59	male	3.8	125	229	164.8	197	463.2	284.2	37	11	0.93
case5	25	male	2.92	102	49	169.1	335	346.5	1044	20.6	14.2	1.21
case6	73	female	3.57	110	84	40.4	68.4	22.4	52	29.5	15	1.25
case7	58	female	4.32	139	124	50.5	55.3	43.7	78.1	39.8	17	1.46
case8	56	female	5.29	165	103	44.7	63.5	71.3	78.2	33.2	15.5	1.31
case9	48	female	2.76	89	249	21.5	31.5	47.9	732.7	31.4	12.6	1.1
case10	61	female	5.04	135	136	29.8	52.5	101.5	85	31.1	15.1	1.3
	P=0.8	P=0.076	P=0.79	P=0.83	P=0.658	P=0.18	P=0.16	P=0.23	P=0.8	P=0.28	P=0.5	P=0.54

Raw data for Figure 4

	admission	preoperat	The first month after TIPS	The second month after TIPS
Case7	43.7	46	56	46.3
Case8	71.3	138	272.5	100.6
Case9	47.9	47.9	51.4	22.6
Case1	101.5	105.8	113.3	58.9
mean	66.1	84.425		
stanc	26.541	45.232		

BUN	CR	Child –Pug	MELD
5.23	57.5	6	6
4.78	65	8	15
6.96	76.2	11	18
5.16	80	7	19
8.86	143	10	25
5.8	63.8	8	10
7.26	102	7	16
4.64	56.2	10	15
7.27	84	9	11
11.3	107	10	18
P=0.3	P=0.7[illegible]	P=0.5[illegible]	P=0.89

Interventional data of 4 patients receiving TIPS

Case 7

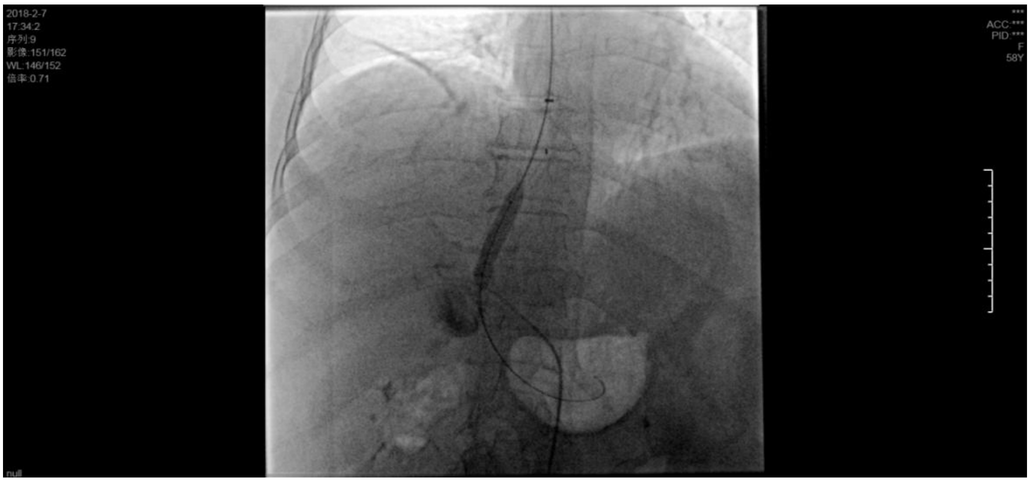

Figure 1　After the portal vein was punctured from the hepatic vein, the puncture tract was expanded with a balloon.This is a picture of balloon expansion.

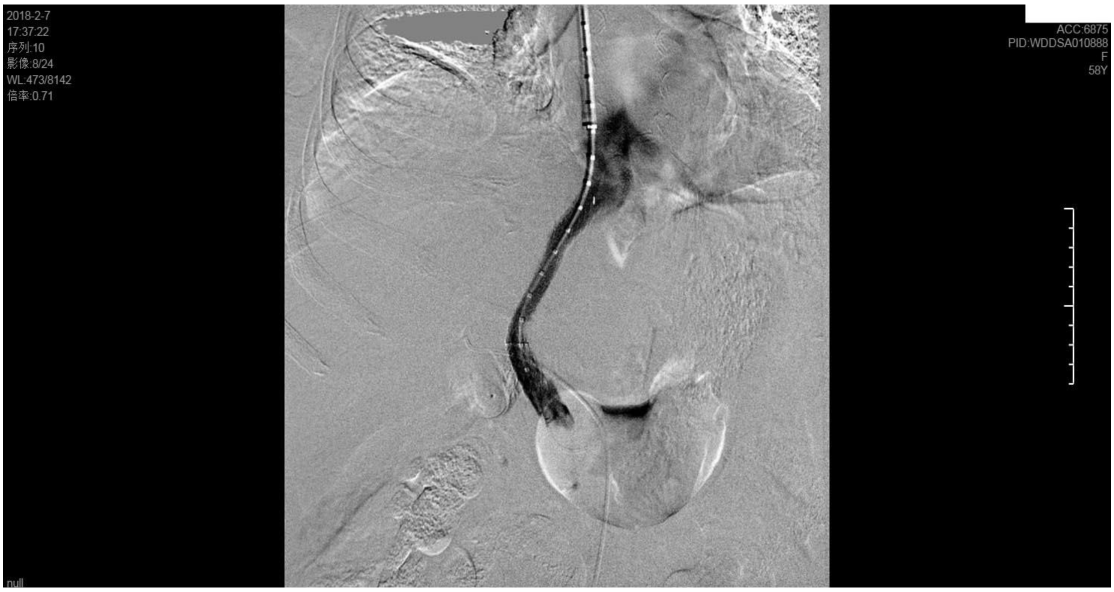

Figure 2　The stent was released in the puncture channel between hepatic vein and portal vein. After release, angiography was performed to determine whether there was stenosis in the stent

Interventional diagnosis report(case7)(chinese version)

武汉市中心医院

放射介入诊断报告单

影像号：WDDSA010888

姓　名：　　性别：女　　年龄：58岁　　检查时间：2018-02-07

病历号：[illegible]　　病区：消化内科二病区（后湖）　　床号：2

造影图像显示：

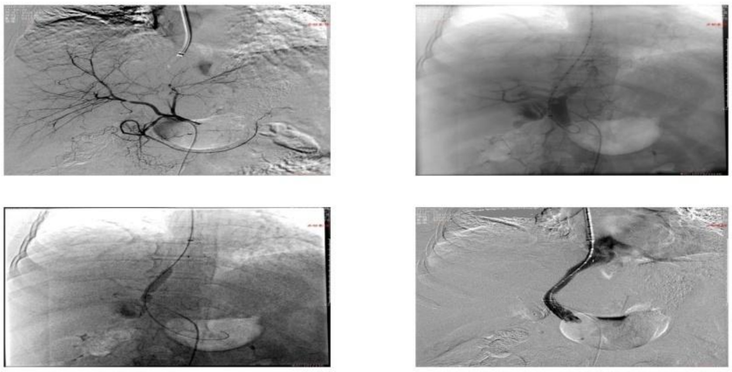

1. 患者仰卧，腹股沟及右颈部消毒、铺巾；局麻后，穿刺右侧股动脉置入5F导管鞘，选用5F“RH”导管插管肝动脉及门静脉间接造影。
2. 使用RUPS 100系统穿刺门静脉成功后，造影见胃底静脉曲张，门静脉主干通畅，测量门脉压力约为35cmH2O。
3. 交换超硬导丝，引入6mm×60mm球囊预扩张穿刺道，置入戈尔8mm×80mm+20mm支架，支架覆盖肝静脉至门静脉主干，再引入8mm×60mm球囊后扩张，建立下腔静脉一门静脉分流道。经导管鞘造影示分流道通畅，测量门脉压力为20cmH2O。
4. 拔除右侧颈静脉及右侧股动脉鞘管，局部无菌包扎固定，结束手术，术程顺利，患者生命体征平稳，术后安返病房。

诊断：

门脉高压；胃底静脉曲张。

报告医师：郑卫华 郑卫华　报告时间：2018-02-08 09:12:40

Case 8

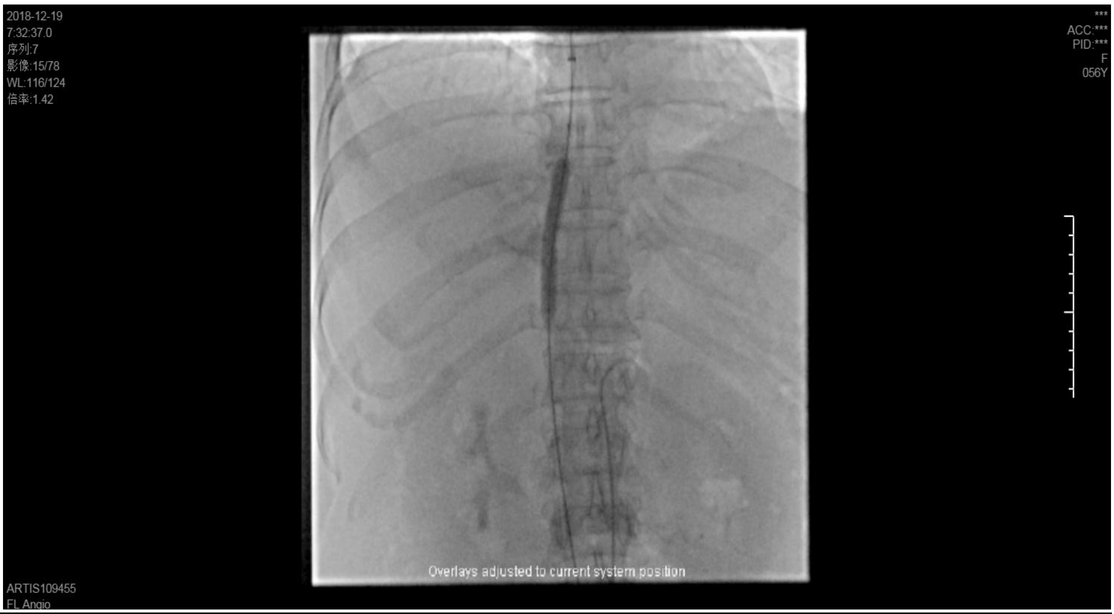

Figure 3 After successful puncture of the portal vein,the puncture tract was expanded with a balloon. In front of the vertebral body, the black part was the balloon.

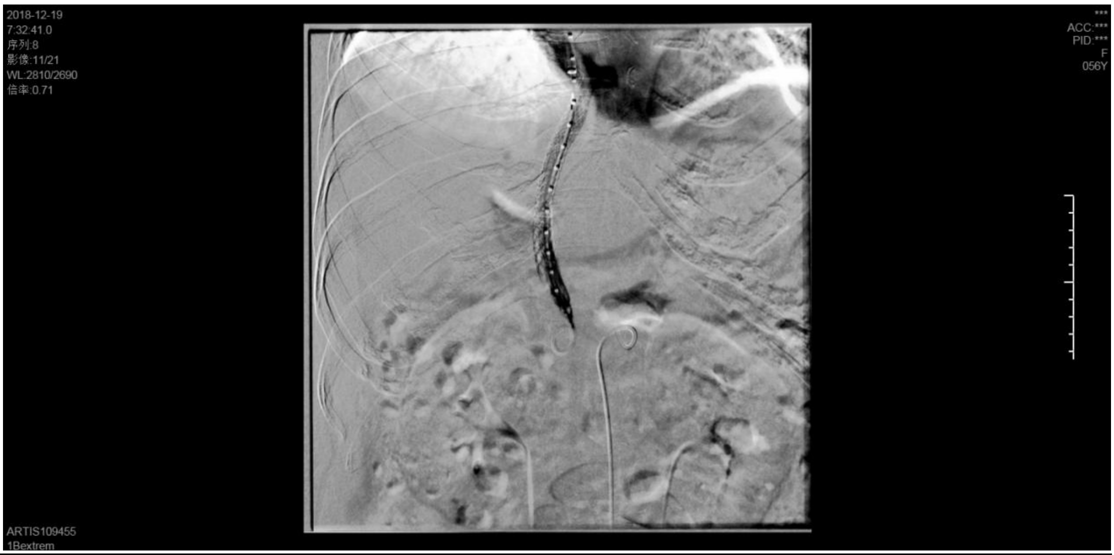

Figure 4 After the stent was released,the portal venography was completed, no filling defect was found in the stent.

Interventional diagnosis report(case8) (chinese version)

武汉市中心医院 case 8

放射介入诊断报告单

影像号：

姓　名：	性别： 女	年龄：56岁	检查时间：2018-12-19
病历号：	病区： 消化内科二病区		床号： 21

造影图像显示：

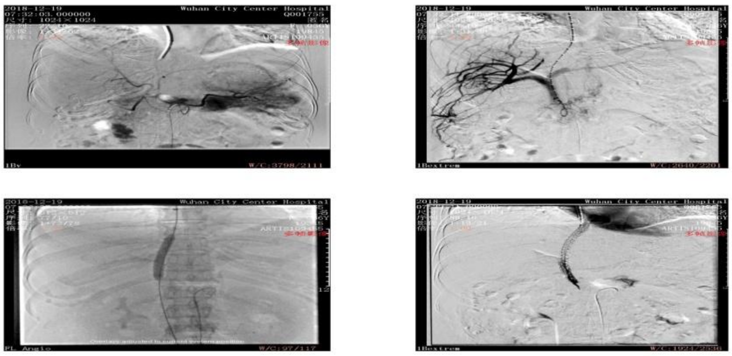

1. 患者仰卧，腹股沟及右颈部消毒、铺巾；局麻后，穿刺右侧股动脉置入5F导管鞘，以5F“RH”导管插管至腹腔干行肝动脉及门脉间接造影。
2. 局麻后，穿刺右侧颈静脉，测量心房压约为17cmH2O。使用RUPS 100系统穿刺门脉成功后，以金标猪尾导管造影见胃底静脉曲张，门脉主干通畅，测量门脉压约为47cmH2O。
3. 交换超硬导丝，引入6mm×60mm球囊预扩张穿刺道，置入戈尔8mm×60mm+20mm支架，支架覆盖肝静脉至门脉主干，再引入8mm×60mm球囊后扩张，建立下腔静脉一门脉分流道。以猪尾导管造影示分流道通畅，测量心房压约为13cmH2O，门脉压约为23cmH2O。
4. 拔除右侧颈静脉及右侧股动脉鞘管，压迫止血并加压包扎，结束手术，术程顺利，患者生命体征平稳，术后安返病房。

诊断：

门脉高压，胃底静脉曲张。

报告医师：郑卫华 郑卫华　报告时间：2018-12-20 09:10:42

Case 9

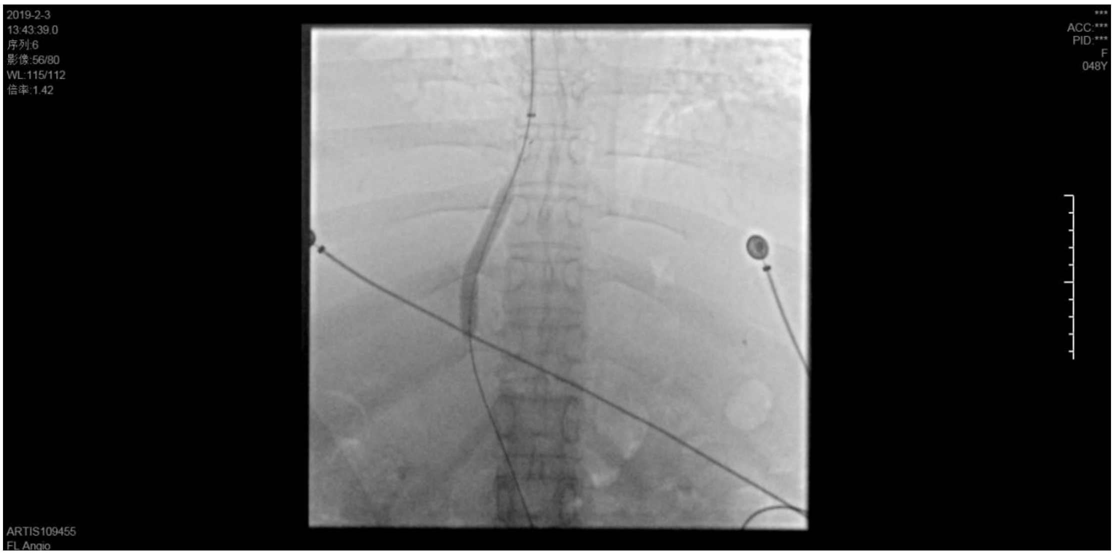

Figure 5　After successful puncture of the portal vein,the puncture tract was expanded with a balloon.

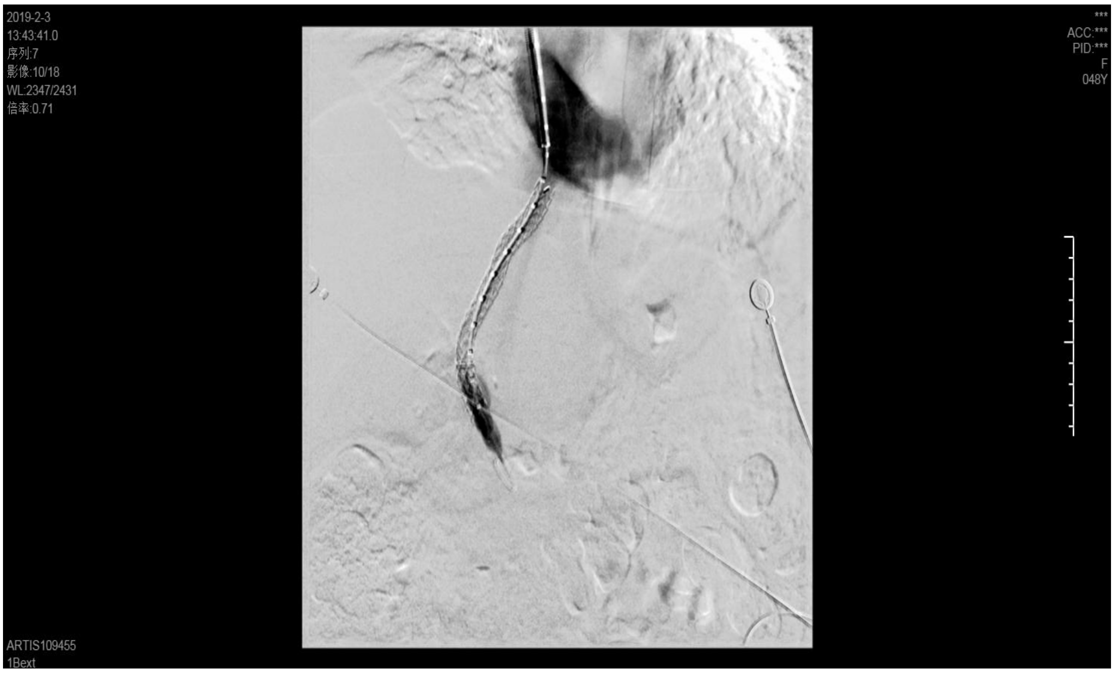

Figure 6　After the stent was released,the portal venography was completed.

Interventional diagnosis report(case9) (chinese version)

武 汉 市 中 心 医 院

放 射 介 入 诊 断 报 告 单

影像号：

姓　名：	性别：女	年龄：48岁	检查时间：2019-02-03
病历号：[illegible]	病区：消化内科二病区		床号：19

造影图像显示：

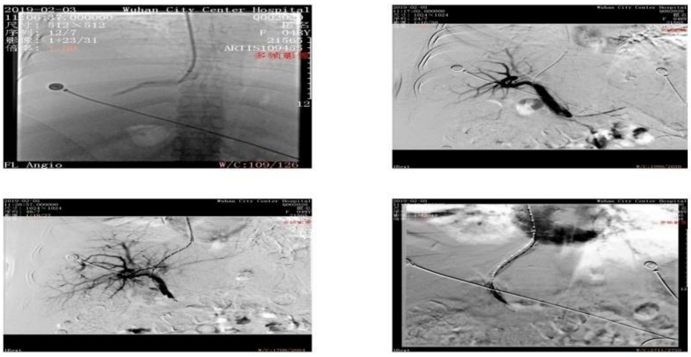

1. 患者仰卧，腹股沟及右颈部消毒、铺巾；局麻后，使用RUPS 100系统穿刺门静脉成功后，造影见门静脉主干显示清晰，但血流缓慢，肝实质弥散不佳，测量门脉压力约为64cmH2O。
2. 交换超硬导丝，引入6mm×60mm球囊预扩张穿刺道，置入戈尔8mm×80mm+20mm支架，支架覆盖肝静脉至门静脉主干，再引入8mm×60mm球囊后扩张，建立下腔静脉一门静脉分流道。
3. 造影示分流道通畅，测量门脉压力为46cmH2O。
4. 术程顺利，术毕拔出颈静脉鞘，加压包扎。

诊断：

肝小静脉闭塞可能，肝硬化，门脉高压，腹水。

报告医师：殷云志　　报告时间：2019-02-03 12:47:29

Case 10

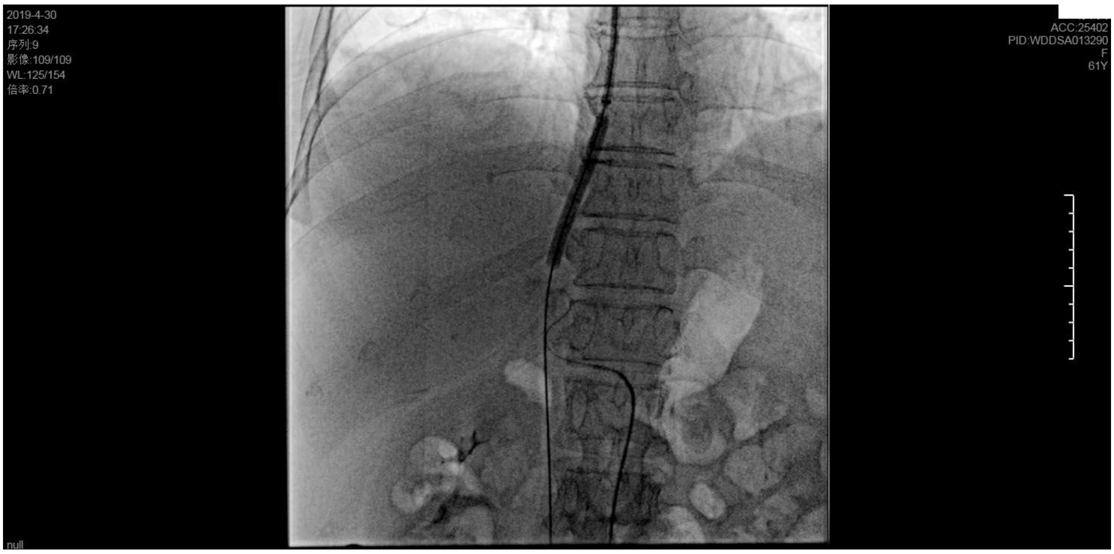

Figure 7 After successful puncture of the portal vein,the puncture tract was expanded with a balloon.

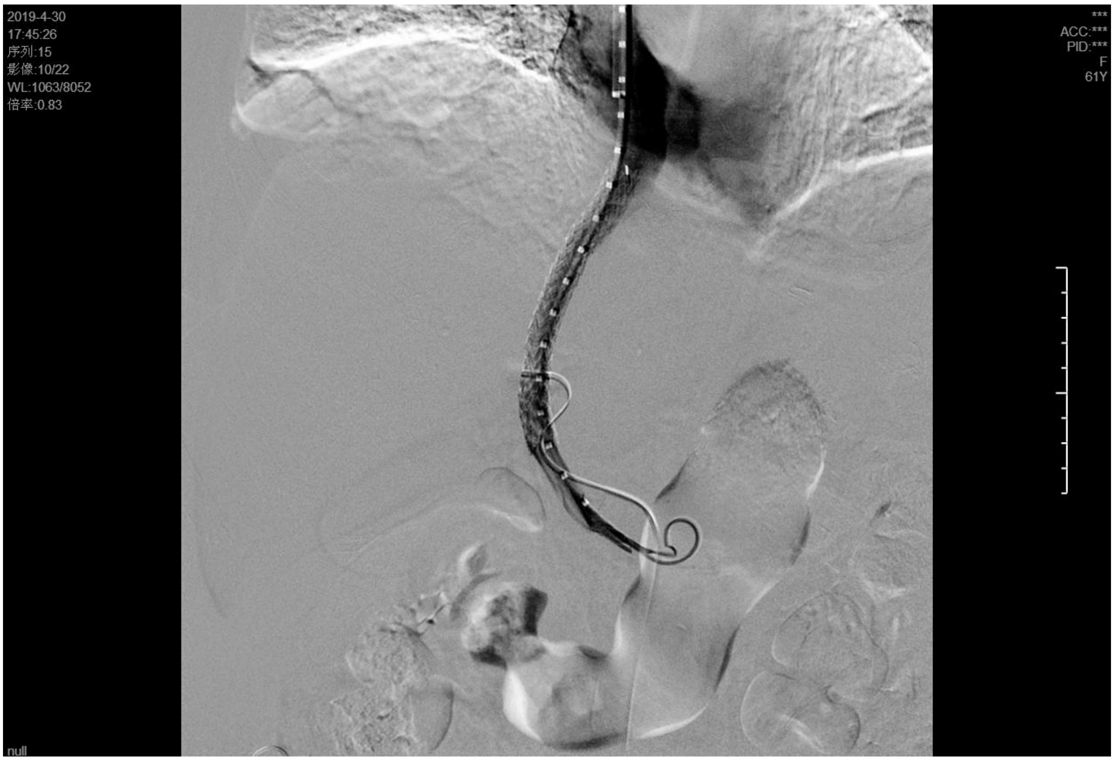

Figure 8 After the stent was released,the portal venography was completed.

Interventional diagnosis report(case10) (chinese version)

武 汉 市 中 心 医 院

放 射 介 入 诊 断 报 告 单

case10

姓　名：　　　性别：女　　年龄：61岁　　检查时间：2019-04-30

病历号：　　　病区：消化科监护室（后湖）　　床号：D-8

造影图像显示：

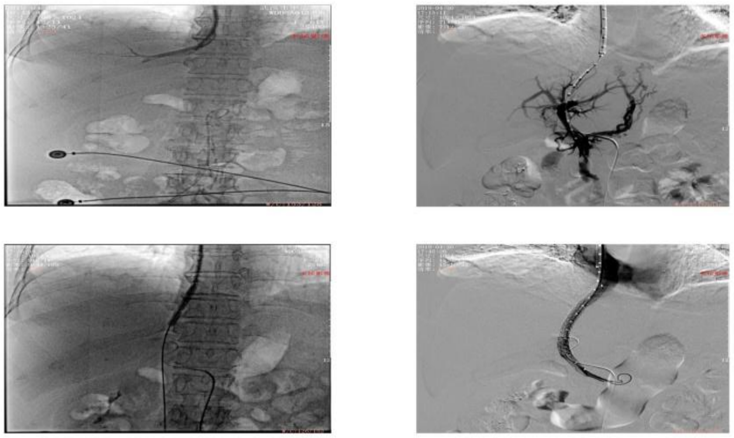

1. 患者仰卧，腹股沟及右颈部消毒、铺巾；局麻后，穿刺右侧颈静脉成功，将导管置于心房水平测压为11cmH2O。
2. 使用RUPS 100系统穿刺门静脉右支成功后，以金标猪尾导管造影见胃底静脉曲张，门静脉右支未显影，主干通畅，测量门脉压力约为40cmH2O。
3. 交换超硬导丝，引入6mm×60mm球囊预扩张穿刺道，置入戈尔8mm×70mm+20mm支架，支架覆盖肝静脉至门静脉主干，再引入8mm×60mm球囊扩张支架，建立下腔静脉一门静脉分流道。造影示分流道通畅，测量门脉压力约21cmH2O；心房水平测压为15cmH2O。
4. 拔除右侧颈静脉鞘管，压迫止血并加压包扎，结束手术，术程顺利，患者生命体征平稳，术后安返病房。

诊断：

门脉高压，胃底静脉曲张。

报告医师：殷云志　　报告时间：2019-04-30 17:50:07
